# Development and assessment of multiplex high resolution melting assay as a tool for rapid single-tube identification of five *Brucella* species

**DOI:** 10.1186/1756-0500-7-903

**Published:** 2014-12-11

**Authors:** Krishna K Gopaul, Jessica Sells, Robin Lee, Stephen M Beckstrom- Sternberg, Jeffrey T Foster, Adrian M Whatmore

**Affiliations:** Department of Bacteriology, Animal and Plant Health Agency, Woodham Lane, New Haw, Addlestone, Surrey, KT15 3NB UK; Center for Microbial Genetics and Genomics, Northern Arizona University, Flagstaff, AZ 86011-4073 USA; MRC Prion Unit, Queen Square House, Queen Square, London, WC1N 3BG UK; Department of Molecular, Cellular, & Biomedical Sciences, University of New Hampshire, College Road, Durham, NH 03824 USA

**Keywords:** High Resolution Melting (HRM), *Brucella*, Species identification, Typing, Real time PCR

## Abstract

**Background:**

The zoonosis brucellosis causes economically significant reproductive problems in livestock and potentially debilitating disease of humans. Although the causative agent, organisms from the genus *Brucella*, can be differentiated into a number of species based on phenotypic characteristics, there are also significant differences in genotype that are concordant with individual species. This paper describes the development of a five target multiplex assay to identify five terrestrial *Brucella* species using real-time polymerase chain reaction (PCR) and subsequent high resolution melt curve analysis. This technology offers a robust and cost effective alternative to previously described hydrolysis-probe Single Nucleotide Polymorphism (SNP)-based species defining assays.

**Results:**

Through the use of *Brucella* whole genome sequencing five species defining SNPs were identified. Individual HRM assays were developed to these target these changes and, following optimisation of primer concentrations, it was possible to multiplex all five assays in a single tube. In a validation exercise using a panel of 135 *Brucella* strains of terrestrial and marine origin, it was possible to distinguish the five target species from the other species within this panel.

**Conclusion:**

The HRM multiplex offers a number of diagnostic advantages over previously described SNP-based typing approaches. Further, and uniquely for HRM, the successful multiplexing of five assays in a single tube allowing differentiation of five *Brucella* species in the diagnostic laboratory in a cost-effective and timely manner is described. However there are possible limitations to using this platform on DNA extractions direct from clinical material.

**Electronic supplementary material:**

The online version of this article (doi:10.1186/1756-0500-7-903) contains supplementary material, which is available to authorized users.

## Background

Brucellosis is a zoonosis of great socio-economic importance that causes reproductive problems including abortions and sterility in large livestock such as cattle, sheep, pigs and goats [1]. In humans, brucellosis can manifest itself in many disparate ailments such as general malaise, fever and arthritis in chronic cases [2]. Transmission from animal to human is facilitated through contact with, or ingestion of, infected material such as abortion tissue or unpasteurised milk from infected animals [2].

The causative agents of brucellosis are organisms from the genus *Brucella*. There are eight recognised species within the genus that have been associated with brucellosis in various terrestrial mammals and at least a further two species associated with marine mammals [3]. However the genus is expanding and recent isolations from baboons, foxes, and frogs suggest that are more groups awaiting description [4–6].

Classically, *Brucella* species are defined through a combination of perceived host specificity and phenotypic characterisation. In this way, *B. abortus* is typically associated with brucellosis in bovines, *B. melitensis* with brucellosis in caprines and ovines, *B. suis* with brucellosis in swine and *B. canis* with canine brucellosis [3]. In ovines, *B. ovis* manifests as ovine epididymitis in rams [1]. However there have been isolations of *Brucella* species outside their perceived hosts, for example, *B. melitensis* infection of cattle being reported [7, 8].

In terms of diagnosis, molecular techniques have been developed for the rapid identification of *Brucella* spp based on genus conserved targets such as *bcsp*31 [9] and IS*711*[10, 11]. As these assays use DNA and not viable bacteria, molecular methods for *Brucella* diagnosis can be more safely used in a wider range of laboratories. Furthermore, there are also molecular tests available that have been developed that can rapidly discriminate to species level from a primary isolation [9, 12–17].

Whilst a number of these tests have been described in the literature, there are two main groups. One group of assays uses specific insertions/deletions identified through genome characterisation of a number of *Brucella* species. These tests include techniques such as AMOS PCR and Bruceladder [12–14] based on a conventional PCR platform as well as assays such as those described by Redkar *et al*., [15] and Probert *et al*., [9] using a real time PCR platform. The second group makes use of single nucleotide polymorphisms (SNPs) identified through Multi-Locus Sequence Analysis (MLSA) using hundreds of *Brucella* strains from all species [18] or through whole genome sequencing. Currently, all recognised and proposed *Brucella* species have been identified with unique MLSA sequence types [4–6, 18] and assays have mostly been developed using real-time PCR platforms and probe based technologies [16, 17]. Although these assays have proven highly effective their implementation is hindered by the expense associated with dual labelled hydrolysis probe multiplexes [16, 17] that make this type of testing potentially difficult to apply in resource limited regions.

One alternative to using hydrolysis probe chemistry is to use melt curves to determine the presence or absence of a target SNP within an otherwise conserved region of sequence [19, 20]. In this scheme, during amplification, an intercalating dye (typically SYBR green) binds to double stranded DNA that forms, generating a fluorescence reading. In the melt cycle, with the increase of temperature, the double stranded product begins to separate and fluorescence drops. The melt peak (which is related to the DNA composition of the product) occurs at a point where 50% of the product population is double stranded and 50% single stranded. Changes in the sequence alter the melt temperature of the target. The major advantage of this methodology over probe-based genotyping is that the chemistry utilised is much cheaper, although this approach has previously suffered from its inability to detect very subtle but significant changes in melt temperatures [21] Nevertheless, recent advances in both dye chemistry and hardware, leading to the development of High Resolution Melt (HRM) curve analysis, facilitates detection of much smaller differences in temperature than previously achieved [22]. Indeed, HRM as a tool for genotyping has been shown to be of great utility [23] not only for bacteria [24, 25] but also for viruses [26] and eukaryote parasites [27]. Furthermore, with pathogen detection and genotyping, there are many examples in the literature of the application of HRM for human genetics to characterise genetic variation linked with various cancers [28, 29] and other ailments [30, 31].

One previous study has described the use of HRM for *Brucella* species identification [32]. However, as in the case of the majority of other publications using HRM for genotyping, this previous study described using one or a number of singular reactions for differentiation. This type of approach in turn reduces the throughput of the system making it less attractive for implementation. Therefore the intention of this study was not only to develop HRM assays as an alternative means of SNP-based *Brucella* species determination but to also combine HRM assays in one tube to improve throughput. To this end this paper describes the creation of a quintuplex test that can rapidly, cheaply and unambiguously define strain identity for five terrestrial *Brucella* species of agro-economic importance; *B. abortus*, *B. canis, B. melitensis*, *B. ovis* and *B. suis*. Of these five species *B. melitensis, B. abortus* and *B. suis* are the species associated with most economically significant animal and human disease [1]. Concerning the remaining two species, *B. canis* infects dogs and is occasional cause of human infection whilst *B. ovis* is a minor and far less-widely distributed animal pathogen.

## Results

### Identification of target SNPs

From multi-locus sequence analysis work described previously a number of SNPs that define particular *Brucella* species have been identified [18]. This extensive work examining a spatially and temporally diverse panel of >700 *Brucella* strains of all *Brucella* species provided the basis for the subsequent multiple outcome species defining assays based on Minor Groove Binding (MGB) TaqMan® probe chemistry [17]. It was therefore initially thought that the same targets could be as the basis of the HRM assays. However using the selection criteria described in the methods section, it was found that none of the previously used targets were suitable markers for use in HRM assays.

It was therefore decided to make use of the expanding *Brucella* whole genome sequence (WGS) project database, hosted by the Broad Institute, Massachusetts, USA. Although this database is not currently as diverse and large as the in-house MLSA, it does none the less provide enough variation (in terms of *Brucella* species) to identify potential markers. Through alignments of 35 *Brucella* spp genome sequences available from the WGS project in March 2010 (Table 1), potential canonical SNPs for the determination of the five targeted *Brucella* species were identified based on location in *B. melitensis* 16 M. Using these data, regions flanking these SNPs were identified and tested *in silico* for secondary structure and melt to see if they met the required criteria. Primers for those regions deemed useful were then designed and tested on a small panel of isolates comprising the type strains for *B. abortus*, *B. melitensis*, *B. ovis* and *B. canis* (Table 2).

**Table 1:** **A breakdown of the 35** ***Brucella*** **genomes used for SNP discovery of species specific targets**

***Brucella*** species	Number of complete genomes available
*B. abortus*	9
*B. canis*	1
*B. ceti*	5
*B. inopinata*	1
*B. melitensis*	4
*B. microti*	1
*B. neotomae*	1
*B. ovis*	1
*B. pinnipedialis*	3
*B. suis*	5
Atypical *Brucella*	4

**Table 2:** **Targets, primer sequences and final concentrations for each of the species-specific quintuplex HRM assays**

***Brucella*** species	Forward primer sequence	Reverse primer sequence	Final concentration of primer pair in quintuplex reaction (μM)	Product size (bp)	Gene target (based on ***B. abortus*** 9-941)
***B. abortus***	5′-GCCCCTCCTTCTTGTAATCA-3′	5′-ACCATGAAGAAAGCGCGTAT-3′	1.25 μM	75	BruAb1_0395Hypothetical protein
***B. melitensis***	5′-ACAAGCTGACGAAGGACCAT-3′	5′-CCCGTATAGGAGTGGATCGT- 3′	0.5 μM	135	BruAb1_1713 glyceraldehyde-3-phosphate dehydrogenase
***B. ovis***	5′-CCGGTCAAGTTCAATCACG-3′	5′-GCTGGAAATGCTCTATTACTC-3′	1 μM	66	BruAb1_1179 Hypothetical protein
***B. suis***	5′-CTGGCGGAAAAGGATTTGAT-3′	5′-AATCACGACAAACCACAGCA-3′	1.125 μM	89	BruAb1_1338 Sugar ABC transporter, permeaseprotein
***B. canis***	5′-CCCCCGTCAATTCCTGCCGAA-3′	5′-CCCCCGTGGCCTGGTCGAGAT-3′	0.25 μM	79	BruAb2_1115 Transcriptional regulator (GntR Family)

### Titration work

**Figure 1:**
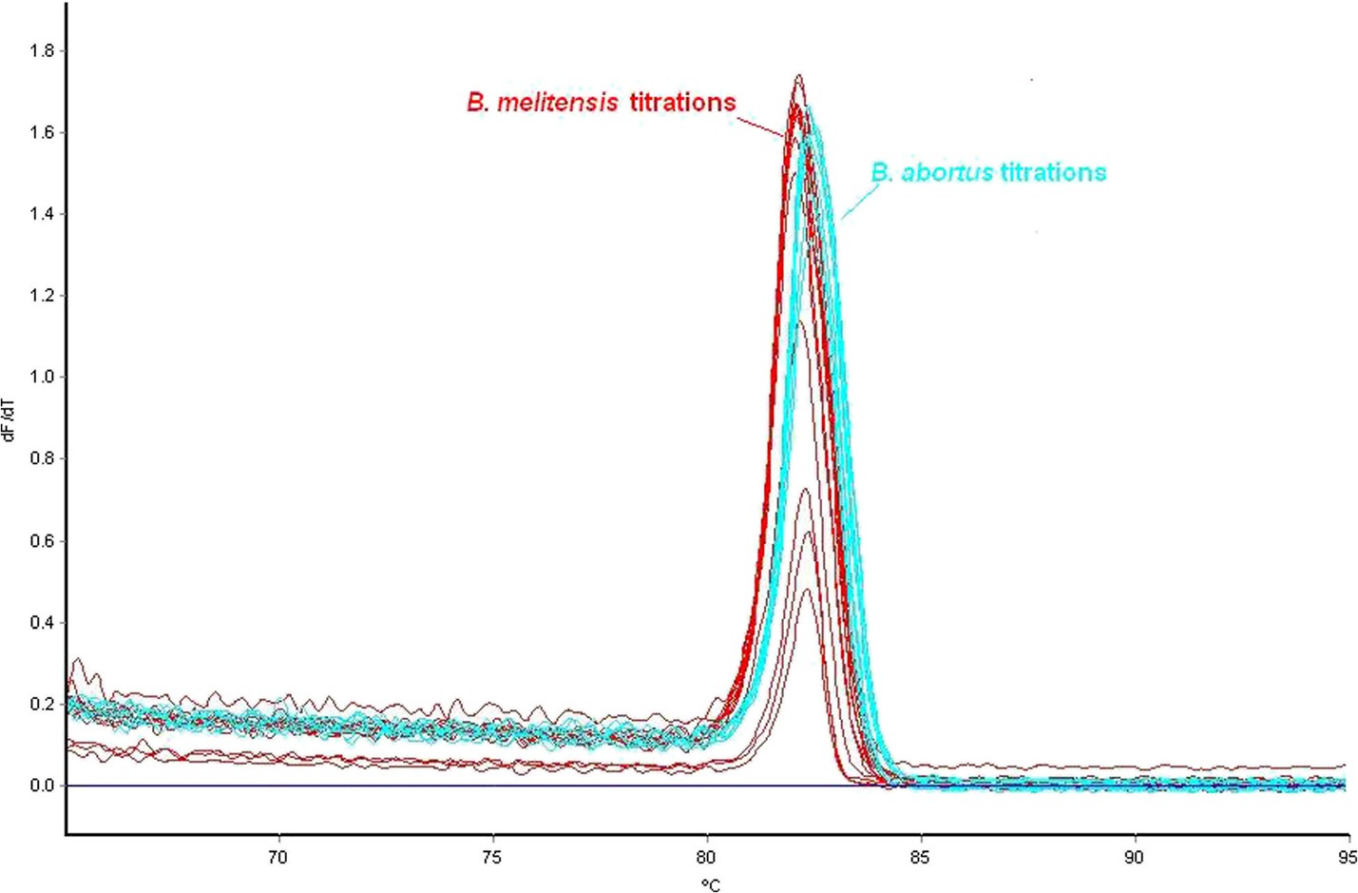
**Melt peaks of one HRM assay with titrations of target and non target DNA.** The results of a titration range of 1 ng-100 fg genomic DNA from *B. melitensis* 16 M (red) and *B. abortus* 544 (blue) using the *B. melitensis* HRM assay and the negative first derivative melt peak data. As shown, whilst the peak size does vary with the concentration of DNA, there is clear differentiation between the two species.

**Figure 2:**
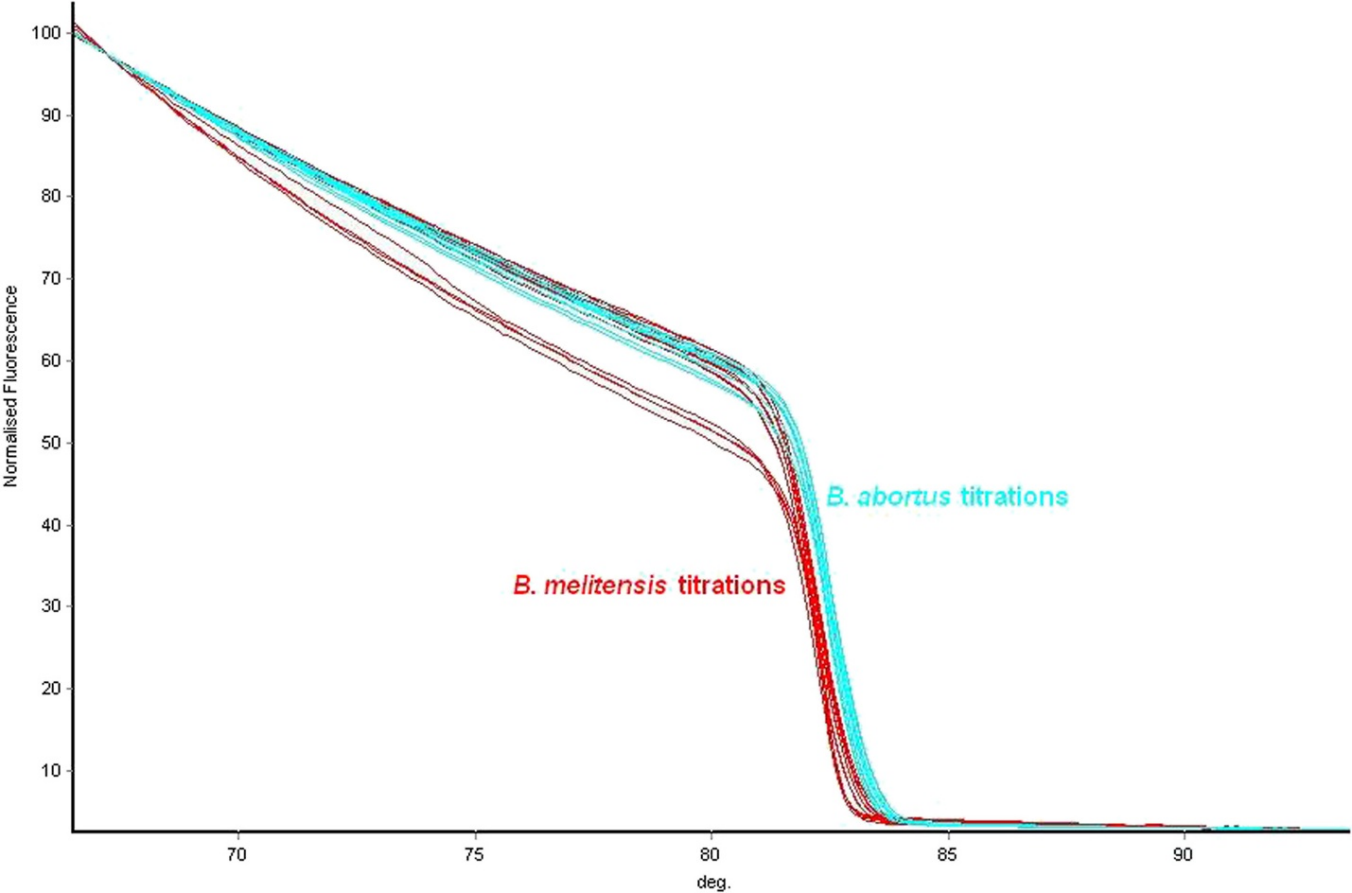
**Melt curves of one HRM assay with titrations of target and non target DNA.** The results of a titration range of 1 ng-100 fg genomic DNA from *B. melitensis* 16 M (red) and *B. abortus* 544 (blue) using the *B. melitensis* HRM assay and the HRM melt curves directly. Curves move from right to left with decreasing DNA concentration. As shown, the curves generated by very low concentrations of *B. abortus* DNA are very close to the curves generated by high concentrations of *B. melitensis* DNA and could be misidentified through this association.

**Figure 3:**
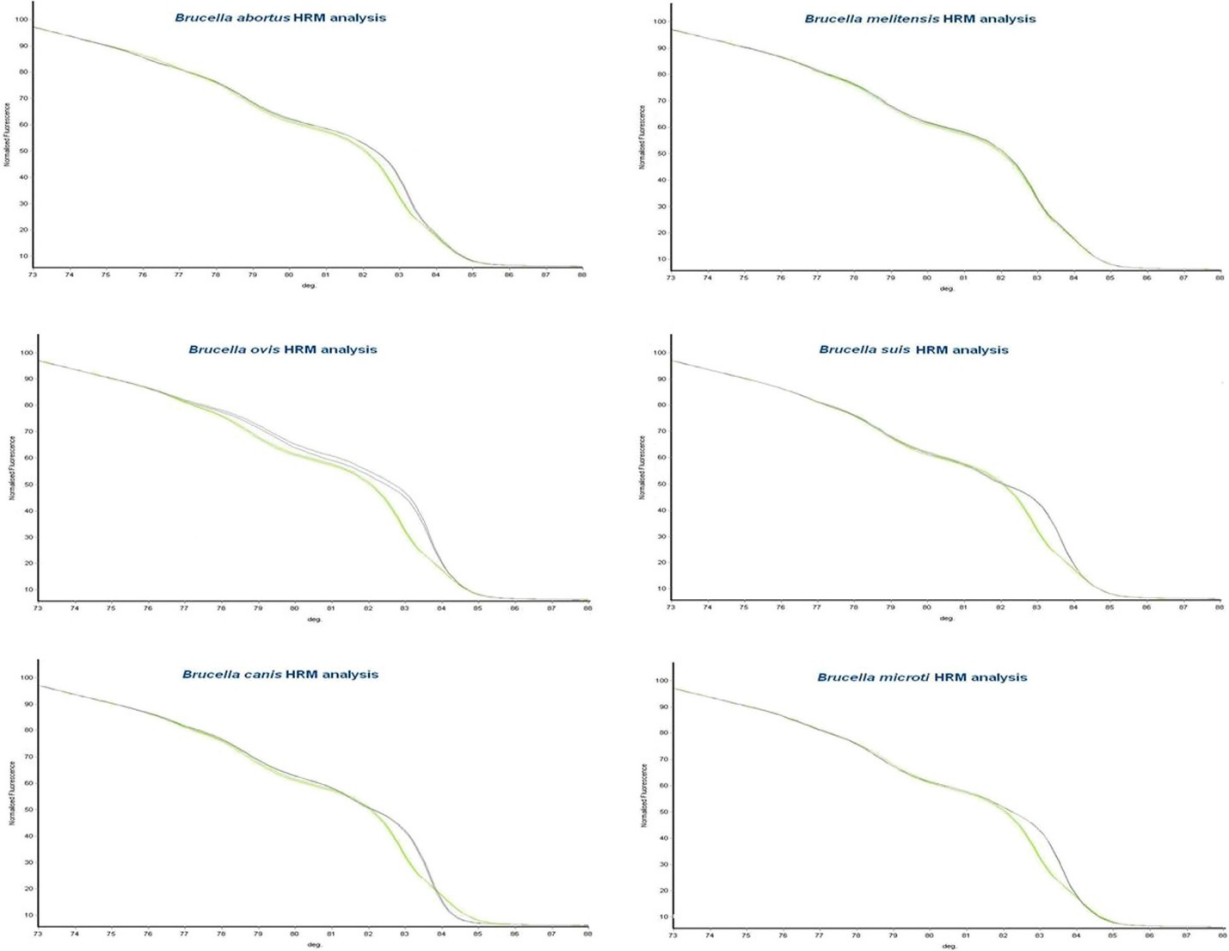
**Determination of species identity based on melt curve kinetics.** This figure gives an example of the diagnostic analysis of an unknown sample (green curve) comparing melt curves with curves generated in the quintuplex assay by representative strains of known *Brucella* species (grey curves). Species identification is based on “best match” with the curves in this case clearly indicating that the query strain is *B. melitensis*. Note that curves generated by *B. microti* isolates*,* not a target species for this assay, are shown to illustrate that a novel trace is generated for species out-with the target scope.

**Figure 4:**
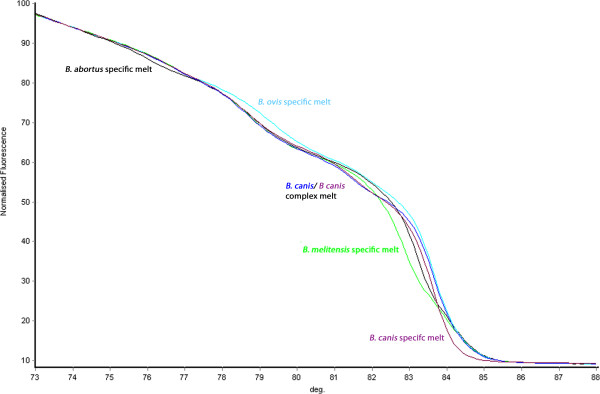
**Melt curve kinetics for quintuplex target species.** Melt profiles illustrating the unique HRM curves generated by the quintuplex for *B. abortus* (black), *B. melitensis* (green), *B. ovis* (light blue), *B. suis* (dark blue) and *B. canis* (purple). Each region of differentiation is highlighted.

To determine the sensitivity of discrimination of HRM assays, titrations of the genomic DNA from the five target *Brucella* species were prepared and tested. Through titrations it was determined that the limit for reproducible discrimination of the five individual HRM assays was 100 fg (data not shown). It was noted that whilst using the negative first derivative of the data, there was no difference in the positioning of melt peaks with changing DNA concentration (Figure 1), there were changes in the kinetics of the melt curve with DNA concentration (Figure 2). As interpretation of the quintuplex was performed visually (Figures 3 and 4), comparing the curve dynamic of the sample with that of a known control on the same run, low concentrations of non-target *Brucella* could be misinterpreted as high concentrations of the target species. In a review of HRM, Reed *et al.*[33] mentioned that interpretation of HRM data was better achieved through the comparison of melt curves rather than from the first derivative melt peak which was subject to “data smoothing”. It was for this reason that the decision was made to standardise the amount of DNA tested to 1 ng/μl in subsequent work.

### Development and validation of the quintuplex

**Figure 5:**
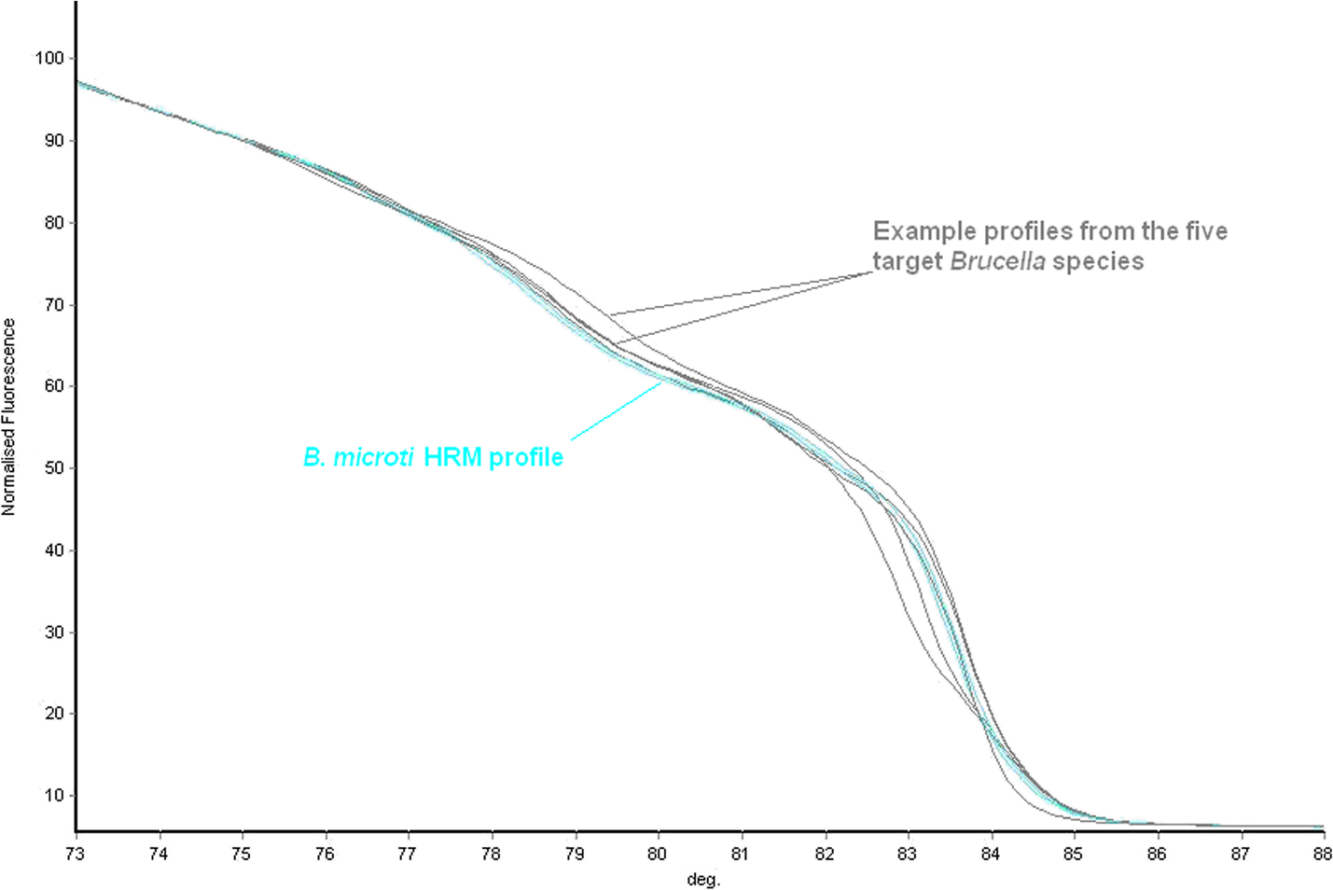
**Melt curve kinetics for quintuplex non target species.** This figure illustrates how a *Brucella* isolate that is not a member of one of the five major species would be excluded by this assay. The curves in grey are traces from the five species targeted by the quintuplex whilst the traces in blue represent a non-target *Brucella* species (in this case *B. microti*). The blue traces are not congruent with any of the grey traces indicting that the sample tested represents a species not included in this assay.

Results of individual reactions for the five species-defining assays demonstrated that the technique was promising, although the issue of change in melt dynamic with the variation in the amount of template added was of concern. However, it was the intention from the conception of this work to develop a species-defining quintuplex. This in turn meant that there had to be 10 distinct melting temperatures to account for each species specific and non-species specific melt. To do this, the melting temperatures of the products of each assay were manipulated to defined melt temperatures by adjusting product size to ensure no overlap between each individual melt peak that would render sample identification impossible. The concentrations of primers added to the primer mix were meticulously optimised to ensure high quality and equally sized melt curves were generated for each species-defining assay in the quintuplex. It was observed that each of the species tested produced a distinct curve profile and that those profiles of isolates not belonging to one of the included five *Brucella* species, such as *B. microti,* are also distinct (Figures 3 and 5).

In the validation of the quintuplex assay, highly purified DNA from 135 isolates that had previously been identified using both classical biotyping and the TaqMan probe based real-time PCR approach (Table 3 for panel breakdown and Additional file 1 for complete strain identity) were tested with the HRM multiplex assays. These isolates were chosen to reflect the genetic diversity of the *Brucella* species in question based on previous MLSA studies [18]. The results of the HRM work were that all 135 samples tested gave species identification congruent with phenotypic and real-time PCR testing [17] (data not shown).

**Table 3:** **A breakdown of the 135** ***Brucella*** **strains used in the validation of the HRM quintuplex**

***Brucella*** species/type	Number of strains tested
*B. abortus*	23
*B. canis*	9
*B. ceti*	10
*B. inopinata*	1
*B. melitensis*	47
*B. microti*	1
*B. neotomae*	1
*B. ovis*	20
*B. pinnipedialis*	9
*B. suis*	13
Atypical *Brucella*	1
**Total**	**135**

### Specificity testing of *Brucella*spp assays

To determine the specificity of published *Brucella* genus molecular diagnostics in an expanding *Brucellaceae* family, two real time PCR assays based on the IS*711* target [10, 11] and one based on the *bcsp*31 target [9] were used to test a panel of 23 non- *Brucella Brucellaceae* (Table 4). It was found that there were no false positive results generated with any of the three assays generated (data not shown), proving that these assays were specific to *Brucella* genus organisms within the *Brucellaceae* family.

**Table 4:** **A list of non-** ***Brucella Brucellaceae*** **used to determine the specificity of three published** ***Brucella*** **spp assays**

Name	Strain number
*Ochrobactrum anthropi*	LMG 3331
*Ochrobactrum ciceri*	DSM 22292
*Ochrobactrum cytisi*	DSM 19778
*Ochrobactrum daejeonense*	JCM 16234
*Ochrobactrum gallinifaecis*	DSM 15295
*Ochrobactrum grignonense*	LMG 18954
*Ochrobactrum haematophilum*	CIP 109452
*Ochrobactrum intermedium*	LMG 3301
*Ochrobactrum lupini*	DSM 16930
*Ochrobactrum oryzae*	DSM 17471
*Ochrobactrum pecoris*	CCUG 60088
*Ochrobactrum pituitosum*	DSM 22207
*Ochrobactrum pseudogrignonense*	CIP 109451
*Ochrobactrum pseudintermedium*	DSM 17490
*Ochrobactrum rhizosphaerae*	DSM 19824
*Ochrobactrum thiophenivorans*	DSM 7216
*Ochrobactrum tritici*	LMG18957
*Paenochrobactrum gallinarii*	CCUG 57736
*Paenochrobactrum glaciei*	JCM 15115
*Pseudochrobactrum asaccharolyticum*	CCUG 46016
*Pseudochrobactrum kiredijianiae*	DSM 19762
*Pseudochrobactrum lubricatis*	CCUG 56963
*Pseudochrobactrum saccharolyticum*	CCUG 33852

## Discussion

### Assay performance

From the testing of 135 *Brucella* isolates, it was observed that the HRM targets and assays correctly identified all isolates to the corresponding species, or in the cases of *B. ceti*, *B. inopinata*, *B. microti, B. neotomae*, and *B. pinnipedialis* as not being members of one of the species included in the assay. These findings were encouraging as although SNPs derived through *Brucella* MLSA work [18] had been rigorously tested with the sequencing of hundreds of *Brucella* strains, the SNPs identified by whole genome sequencing had been selected after comparison with a much smaller group of 35 sequenced *Brucella* genomes. Although the test panel chosen for this work is relatively small, the selection and testing of a wide number of *Brucella* MLSA sequence types shows the robustness of the SNPs chosen.

The HRM method offers a simple solution to SNP-based genotyping, with little downstream processing and a closed tube excluding the hazards of product manipulation and possible subsequent cross-contamination. In terms of analysis, data acquisition is rapid with a melt only taking a few minutes post amplification. Data analysis is straightforward with species assignment based on a visual comparison of curve kinetics with reference strains on the same run (Figure 3). Although there is an upfront hardware cost, once purchased reagent prices are lower than those associated with Taqman-probe based SNP typing. In addition, the testing for five *Brucella* species in one tube gives equivalent resolution but at five times the throughput of individual assays such as those based on the use of hydrolysis probes [17, 18]. This is yet another cost reduction measure presented by this HRM assay that makes the use of SNPs for the identification of *Brucella* sequences more attractive.

In this study, the focus has been on the development of a quintuplex focusing on five species that commonly infect animals of agro-economic importance. However, this work should also been seen as a proof of concept, highlighting the possibility of multiplexing several SNP-based species defining assays for better throughput and reduced costs. Although other studies have also demonstrated the possibility of multiplexing HRM assays in triplex and quadruplex format [34, 35], this present work is an example where five individual bacterial targets have been characterised simultaneously in a single tube.

### Comparison with other *Brucella*HRM assays

As mentioned earlier, the *Brucella* quintuplex HRM is not the first example of the application of HRM for the identification of *Brucella* species. Work published by Winchell *et al.,*[32] described the application of a number of assays to comprehensively cover all known *Brucella* species with the exception of *B. inopinata*. Further, the incorporation of an assay for the direct identification of members of the *Brucella* genus allows for direct identification from isolation, something that is not available using the *Brucella* quintuplex. However, in defence of the quintuplex, it should be stated that there are already a number of *Brucella* real time PCR diagnostic assays available that have been validated on large panels of non- *Brucella* organisms [9–11]. Any of these assays could be used as a rapid screen to identify *Brucella* spp organisms. Indeed, work undertaken within this department using a number of non-*Brucella Brucellaceae* (Table 3) and three published assays based on IS*711*[10, 11] and *bcsp*31 [9] has shown that these assays are *Brucella* spp specific even in light of an expanding *Brucellaceae* genus (data not shown) and thus, these assays could be used as an initial screen for *Brucella* organisms prior to species differentiation.

In terms of targets, whilst both schemes use individual SNPs for each species, the Winchell paper also includes one target for two species; the gene *glk* for *B. abortus* and *B. ovis*[32]. From MLSA data it can be seen that whilst a majority of *B. abortus* strains tested do contain the SNP identified as *B. abortus* specific by Winchell *et al.,*[32] a significant subset of isolates of African origin, including *B. abortus* biovar 3 type strain and field isolations of *B. abortus* biovars 3 and 6 have the alternate state SNP [18]. Further, to compound the issue, the SNP identified by Winchell *et al.*[32], as specific to *B. neotomae* is also found in the *B. abortus* biovar 3 type strain, leading to a possible misidentification with this HRM assay, Winchell *et al.*[32] do not state whether the *B. abortus* biovar 3 strain used was the type strain or a field isolation but this one example highlights a potential limitation in the universal applicability of this scheme.

Another major difference between the previously published *Brucella* HRM assay and the proposed quintuplex is the used of an insertion rather than a SNP to positively identify *B. suis* isolates in the former. From the SNPs identified in the MLSA work published by Whatmore *et al.,*[18] it was not possible to identify a *B. suis* specific SNP that positively identified all five *B. suis* biovars but excluded *B. canis*. This reflects the phylogenetic placement of *B. canis* as a terminal node within the *B. suis* clade. However, the authors also mention that they could not differentiate *B. suis* biovar 4 from *B. canis* isolates, meaning that there could be misidentifications between certain *B. suis* and *B. canis* isolations. In comparison, the *Brucella* quintuplex uses SNPs derived from whole genome sequencing for the identification of all *B. suis* biovars and it has been shown to identify type strains from all five biovars in addition to clinical isolations. This is an improvement over previous hydrolysis probe based *Brucella* SNP assays that could not identify *B. suis* biovar 5. Previous molecular studies using variable number tandem repeat (VNTR) analysis and MLSA had suggested that *B. suis* biovar 5 might be considered a novel *Brucella* species [18, 36] although recent WGS analysis does confirm the placement of *B. suis* biovar 5 as a very early branch in the *B. suis*/*B. canis* clade [37, 38]. Though *B. canis* shares this SNP, the quintuplex makes use of a unique *B. canis* SNP to separate this species, and despite this being an indirect testing algorithm it at least excludes the possibility of samples being misidentified.

### Issues with the use of HRM

Although the reagent costs of running HRM assays are significantly cheaper than that of the equivalent hydrolysis probe real-time PCR tests, there are some issues that could potentially diminish the usefulness HRM for typing. Although the design process for HRM primers is straightforward, target choice is hampered by the need for minimum secondary structure. Evidence of this has been seen early in this current study with the inability to design HRM assays to targets previously used for hydrolysis probe based assays [17].

Further, this technique cannot identify the location of target SNP and so if there is a substitution that causes identical change in melt temperature elsewhere in the fragment other than the region of interest, this will be miscalled. Nevertheless, the known lack of genetic diversity in classical *Brucella* species [3] suggests this is unlikely to be an issue for *Brucella* spp HRM assay development.

In this study, it was observed that when titrations of different *Brucella* species were compared the limit of reproducible species identification for each of the five individual HRM assays was 100 fg or approximately 30 bacterial cells. Winchell *et al.,*[32] also found that this level of sensitivity in the majority of assays tested and suggested that there may be a role for this type of testing in the direct typing of *Brucella* species from clinical material. However, whilst the sensitivity of the technique in determining species from purified DNA is clear, there are doubts as to the usefulness of HRM assays where the input DNA is not quantified. In this study, from the tests of titrations of target DNA as well as in other published work on HRM [21, 25, 30], it was shown that melt curve characteristics are affected by the initial concentration of DNA template in the reaction. Another worrying observation reported in relation to the previously published *Brucella* HRM assay was the greater amount of DNA required for reproducible identification using the *B. melitensis* assay [32], intimating that at lower concentrations, incorrect typing results may be obtained. Whilst DNA quantification is straightforward in the case of test application following bacterial isolation, this would be problematic in field samples where the large excess of host DNA makes quantification of pathogen DNA impossible. In contrast, prior DNA quantification is not an issue for SNP based typing using hydrolysis probes. If a sample is positive using *Brucella* spp real time PCR assays, in theory it should be possible to undertake SNP typing using hydrolysis probes.

A further drawback is the fact that the melting temperature of DNA in this technique is not only dependant on the concentration of template pre-amplification but also on ionic conditions [21]. In turn this means not only does the sample need quantification pre-amplification but that the extraction methods and how the sample storage matrices. This may have implications for the type of sample that can be tested using this technique, with crude extractions (e.g. heat inactivated cells) possibly being problematic. However, this can be rectified by running these extractions thorough a commercial DNA extraction kit.

In spite of these issues, the demonstration of the possibility to multiplex SNP-based HRM assays is a major selling point for this technology. In contrast, the rigidness of the two fluorophore hydrolysis probe method only allows for two possible outcomes per reaction; does this sample possess or lack the SNP that is targeted in this reaction. However, whilst the HRM method does improve throughput by the combination of assays, it should be noted that this method described in this paper requires the use of known *Brucella* strains in every run to allow for comparison with unknown samples.

One final issue in the use of HRM is the ability to multiplex reactions to allow for “one tube” species identification on a real-time PCR platform. In this study, five targets were combined to allow for the identification of five terrestrial *Brucella* species. This was done through the variation of the product sizes and primer concentrations for each of the assays used. There are SNPs that can identify all recognised *Brucella* species (currently ten in total) and vaccine strains [18, 39], A. M. Whatmore and M. S. Koylass unpublished data]. Nevertheless, although it has been suggested that larger multiplex reactions may be possible [35], given the way the data is currently analysed in the quintuplex, the addition of further *Brucella* targets for vaccines and other species to the described assay may make the interpretation of data generated more difficult and thus, full characterisation may require the running of several HRM multiplex reactions.

## Conclusion

In conclusion, the HRM technique is a highly accurate method for typing *Brucella* to species level based on single diagnostic SNPs. It is a simple and rapid closed tube test with minimal risks of cross-contamination. Assay design is straight forward using freeware tools and the chemistry utilised is much cheaper to run than the chemistry utilised by hydrolysis-probe mediated SNP identification, allowing for wider use of *Brucella* SNPs for molecular-based species identification. In this study, through optimisation of primer concentrations, it was possible to multiplex five *Brucella* species-identifying HRM assays in one tube allowed for a much higher throughput than the equivalent assays based on hydrolysis probe chemistry. Although limitations in the number of targets that can be multiplexed in one reaction have been identified, the HRM platform is open and there is the potential to add further *Brucella* SNP-based HRM assays to allow for more comprehensive identification.

However, whilst the quintuplex provides a useful and cheaper alternative to that of hydrolysis probes for real-time SNP typing when applied to bacterial culture material, these techniques may not be appropriate for direct testing from clinical material as there are constraints regarding condition quantification of DNA samples prior to amplification.

## Methods

### SNP discovery and assay development

Data from both the AHVLA *Brucella* MLSA scheme and from an international whole genome sequencing project hosted by the Broad Institute, Cambridge, USA (http://www.broadinstitute.org/annotation/genome/brucella_group/MultiHome.html) were used to identify SNPs (and associated flanking sequence of 100 bp) specific to the five most significant *Brucella* species; *B. abortus*, *B. melitensis*, *B. ovis*, *B. suis* and *B. canis*. The criteria for target choice were the presence of only one known SNP per amplicon, and amplicon size of between 70 bp and 250 bp. Overall amplicon sizes for the different species targets were staggered to allow for subsequent multiplexing of assays. Secondary structure in potential targets was determined using the DINAMelt programme available freely online (http://mfold.rna.albany.edu/?q=DINAMelt) and regions with a change in Gibbs Free energy (ΔG) value > -1 were taken forward and used as the basis for subsequent primer design. Primers were then designed using the Primer 3 software (http://frodo.wi.mit.edu/primer3/). For each target selected, a number of primer combinations were generated by this software, synthesised and tested in simplex reactions.

### HRM reaction set up and analysis

Reactions were run in a volume of 25 μl using Qiagen Type-it® HRM™ PCR mix (Qiagen, Crawley, West Sussex, United Kingdom). Each reaction contained final concentrations of 1x HRM Type-it™ mix (Qiagen, Crawley, West Sussex, United Kingdom) and 0.7 μM forward and reverse primer (for each target). Each sample was tested in duplicate. Reactions were run on a Qiagen Rotorgene-Q 5-plex machine (Qiagen, Crawley, West Sussex, United Kingdom) with temperature cycling parameters for the amplification stage being a hold of 95°C for 5 minutes, followed by 40 cycles of 95°C for 10 seconds, 60°C for 30 seconds, and 72°C for 20 seconds. For the HRM stage, fluorescence recordings were made over the range of 65-95°C by increments of 0.1°C. A normalised graph was generated using normalisation regions of 73-75°C and 86-88°C.

To determine the sensitivity of differentiation, titrations of genomic DNA from four species (*B. abortus*, *B. melitensis*, *B. ovis* and *B. suis*) were prepared from 1 ng/μl to 100 ag/μl and tested with single HRM assays. For all subsequent work DNA samples were quantified and standardised to 1 ng/μl prior to testing.

Assays were first tested individually to determine suitability for further work. After the species specificity of each individual assay had been determined, multiplexing was attempted. With multiplexing of assays, only primer concentrations for the species-specific assays were varied to allow for optimum differentiation of samples from different *Brucella* species. Once concentrations of each assay used were optimised, a panel of DNA isolated from 135 *Brucella* isolates was selected to validate the multiplex (Table 2 for species composition; Additional file 1: Table S1 for further characterisation). The strains used in this study form part of a collection of bacterial isolations held at AHVLA. As only bacterial DNA from this collection was used as testing material, this work did not require approval through the AHVLA ethics committee. Strain identity was assigned based on the visual similarity of the melt curve generated by the unknown to melt curves generated by reference strains of the five target species. As there was no means of storing these reference curves generated for analysis over different runs it was necessary to include an example of each of the five target species in every run undertaken.

### Set up for *Brucella*spp real time PCR assays

The real time PCR assays used for this section of this study were run as published [9–11] with the following amendments. For DNA template, 2 μl of heat inactivated bacterial culture suspension was used for each of the *Brucellaceae* species tested (Table 4). This material was previously indentified and confirmed as being fit for PCR using 16SrRNA sequencing [40].

## Electronic supplementary material

Additional file 1: **Table showing the test panel of 135** ***Brucella*** **strains used for the validation of HRM quintuplex assay including full strain identity.** (XLS 38 KB)

## Abbreviations

bp: Base pairs
HRM: High resolution melting
MGB: Minor groove binding
MLSA: Multi locus sequence analysis
PCR: Polymerase chain reaction
SPP: Species
SNP: Single nucleotide polymorphism
ST: Sequence type
VNTR: Variable number tandem repeat
WGS: Whole genome sequencing.
